# Chromatin modifications: The driving force of senescence and aging?

**DOI:** 10.18632/aging.100023

**Published:** 2009-02-13

**Authors:** Teresa DiMauro, Gregory David

**Affiliations:** ^1^ Department of Pharmacology, NYU Langone Medical Center, New York, NY 10016, USA; ^2^ NYU Cancer Institute, NYU Langone Medical Center, New York, NY 10016, USA

**Keywords:** chromatin, histone, senescence, heterochromatin, methylation, nuclear organization

## Abstract

An emerging field of investigation in the search for treatment of human disease is the modulation of chromatin modifications. Chromatin modifications impart virtually all processes occurring in the mammalian nucleus, from regulation of transcription to genomic stability and nuclear high order organization. It has been well recognized that, as the mammalian cell ages, its chromatin structure evolves, both at a global level and at specific loci. While these observations are mostly correlative, recent technical developments allowing loss-of-function experiments and genome-wide approaches have permitted the identification of a causal relationship between specific changes in chromatin structure and the aging phenotype. Here we review the evidence pointing to the modulation of chromatin structure as a potential driving force of cellular aging in mammals.

## Introduction

In mammals, aging is a complex phenomenon likely to result from a myriad of molecular changes. Studying mammalian aging experimentally represents an obvious challenge that has hampered our understanding of the molecular mechanisms underlying this process. In culture, cells have a limited proliferative lifespan in which they undergo an irreversible cell cycle arrest known as replicative senescence [1]. Cellular senescence corresponds to an irreversible exit from the cell cycle and can be triggered in primary cells by different stimuli, including expression of activated oncogenes (oncogene-induced senescence), or serial passaging (replicative senescence). Specifically, cellular senescence is believed to occur when cellular growth is promoted while cell cycle progression is inhibited, as observed for example upon strong mitogenic stimuli [2,3]. Irrespective of the stimulus used to drive senescence, senescent cells become flat and enlarged, and are positive for β-galactosidase activity in acidic conditions [4]. Senescence is believed to reflect the aging process at a cellular level, and therefore represent an ideal model system to study the molecular basis of aging. While there has been some question as to whether cellular senescence contributes to organismal aging, it is believed that senescence can lead to the reduction of the regenerative potential of the stem cell pool. Additionally, accumulation of senescent cells in tissues could impair tissue function [5]. Importantly, recent studies using skin biopsies from mammals have shown that senescent cells accumulate, *in vivo*, during aging and can end up representing more than 15% of total cells in aged animals [6]. This study suggests that cellular senescence is a hallmark of organismal aging.

Importantly, chromatin modifications have been shown to play a role in the determination of the senescent phenotype. In eukaryotic cells, DNA is tightly associa-ted with histones as well as non-histones proteins to form the chromatin fiber. Specifically, histones are assembled as octamers composed of two copies of each core histone, H2A, H2B, H3 and H4 to form the nucleosome. Through post-translational modifications and protein recruitment, the nucleosome is an essential component of dynamic chromatin regulation. Histones are subjected to specific modifications including acetylation, methylation, phosphorylation, ubiquitylation, ADP-ribosylation and sumoylation, while DNA itself can be methylated [7,8]. These specific modifications result in higher order chromatin structure and regulate DNA accessibility. Importantly, the combination of different modifications results in specific transcriptional or structural outcomes, underlining the existence of a "histone code" [9,10]. Such a code tremendously enhances the degree of information coded solely by the DNA molecule. Therefore chromatin modifications impact all biological processes related to DNA, including gene expression and genomic stability, both of which are at the nexus of the senescent phenotype. Here we discuss the evidence suggesting that chromatin modifications can be the driving force behind the senescent phenotype and their implications in organismal aging.

### Age related DNA methylation changes

Early experiments in mammalian cells have demonstrated the occurrence of a global decline in DNA methylation in cultured primary fibroblasts from mice, hamsters, and humans compared to their immortalized counterparts. These observations suggested for the first time that replicative senescence may correlate with changes in chromatin structure [11]. Importantly, these results have also been confirmed *in vivo *[12], as the 5-methyldeoxycytidine content in DNA isolated from mouse livers, brains, and small intestinal mucosa significantly decreased as the animals age. The overall decline of methylation results mostly from the loss of DNA methylation at repetitive regions. Repetitive sequences, including satellite repeats at pericentric and centromeric loci, and interspersed repeat sequences, represent about 45% of the mouse genome [13] and are normally highly methylated. It is widely accepted that DNA methylation correlates in most cases with the generation of a repressive chromatin structure, thus preventing potential deleterious recombination within repetitive DNA sequences. Interestingly, these deleterious recombinations accumulate with age, leading to increased incidence of disease including cancer [14], suggesting that the progressive loss of DNA methylation at repeat sequences may account, at least in part, for the accumulation of genomic aberration in aging organisms. In addition, since DNA methylation is normally associated with gene repression, it is conceivable that hypomethylation of these regions results in gene re-expression at these loci.

Despite a global decrease in total DNA methylation, specific sites in the genome become hypermethylated as mammals age [15], suggesting that the enzymatic activity associated with DNA methylation is not entirely impaired during the aging process. This increase in DNA methylation occurs mainly at CpG islands, which are typically unmethylated in normal tissues. However, methylation at discrete CpG islands increases during the aging process [15]. Specifically, the locus encoding the estrogen receptor gene has been shown to become increasingly methylated at CpG islands in human colon in an age dependent fashion [16]. Similarly, genes encoding ribosomal RNA become progressively methylated in rat liver [17]. Finally, multiple tumor suppressor or tumor related genes such as APC, E-cadherin, GSTP1, and p16^INK4A^ have shown different levels of methylation in an age and tissue specific manner [15,18,19].

While the significance of these changes remains unclear, these observations, pointing to the differential modulation of DNA methylation at gene-specific loci with age, are at first glance in agreement with the "redistribution of chromatin modifiers" (or RCM) hypothesis [20]. According to the RCM hypothesis in aging, chromatin modifiers leave their normal target loci as the cell ages, and are relocalized to other sites, thus altering the chromatin structure in two ways. However, the substrate specificity of methyl-transferases makes this unlikely in the case of DNA methylation. Various enzymes account for the methylation events that occur on genomic DNA in mammals. Dnmt1 is responsible for methylating newly replicated DNA and, accordingly, uses hemimethylated DNA as a substrate; it is therefore responsible for maintaining methylation patterns [21]. In contrast to Dnmt1, the related Dnmt3a and Dnmt3b enzymes contribute to *de novo* methylation, in that they can methylate previously unmethylated DNA [22]. Enzymatic assays have revealed a decrease in maintenance methylation concomitant to an increase in *de novo* methylation in senescent human fibroblasts compared to their early passage counterparts [23]. Accordingly, the amount of transcripts corresponding to Dnmt1 decrease significantly as fibroblasts senesce, while Dnmt3b transcription is up-regulated, suggesting the loss of methylation results at least in part from changes in Dnmts transcriptional regulation [24]. Collectively, these observations suggest that during the aging process, a change in the balance between de novo methyl-transferases and maintenance methyl-transferases is likely to contribute to the changes observed in chromatin structure. Whether these changes in DNA methylation directly contribute to cellular aging or represent a mere consequence of this phenotype remains an unresolved issue.

### Histone modifications, histone modifiers, and senescence

As presented above, histones are modified at many residues within the tails or even the globular domains, where different covalent modifications can be found [10,25]. Specific histone modifications have yet to be fully studied and understood in organismal aging. Deciphering the contribution of specific histone modifiers to the aging phenotype is impaired by the usually pleiotropic effects of these enzymes, whose inactivation leads in many cases to cell lethality.

In spite of these impediments, specific histone modifications have been linked to the aging process. For example, trimethylation of histone H4 at lysine 20 (H4K20me3), a hallmark of constitutive heterochromatin, increases in rats livers with age, and is found upregulated in a cellular model of progeria [26,27]. Since genetic inactivation of Suv4-20, a family of enzymes responsible for this modification, results in proliferation defects due to increased sensitivity to DNA damage, the specific function of these enzymes in cellular senescence remains unclear [28]. In addition, the modulation of Suv4-20 methyl-transferase activity or expression level in young versus old cells or tissues has yet to be investigated. By contrast, a direct and well-established function for a histone modifier in senescence is that of the histone methyl-transferase EZH2. As reviewed in [29], the INK4A locus is a major player in the induction of senescence in mammalian cells. Upon replicative or oncogenic stress, the products of this locus, the p19^ARF^ and p16^INK4A^ proteins, accumulate, leading to growth arrest. Recent studies have implicated an EZH2-containing complex (known as PRC2) in the transcriptional repression of the INK4A locus in proliferating cells. Upon signals triggering senescence, EZH2 levels decrease, concomitant with the loss of H3K27me3 mark at the INK4A locus [30]. While these observations definitely implicate a histone methyl-transferase on the induction of a senescence transcriptional program, whether active demethylation on H3K27 is also involved in this process remains unclear [31]. Finally, the histone demethylases KDM2a and KDM2b, which target methylated H3K36, prevent senescence by modulating the p53 and Rb pathway [32,33]. Specifically, overexpression and loss-of-function experiments have shown that this function is accomplished through their ability to demethylate H3K36me histones at the p15^INK4B^ locus, leading to its repression. Together, these data demonstrate that histone modifiers are able to contribute in a gene-specific manner to the induction or prevention of cellular senescence. By contrast, the contribution of chromatin modifiers to cellular senescence and aging as global regulators of chromatin structure remains elusive and awaits genome-wide approaches to definitively ascertain these functions.

Similar to what was reported for DNA methylation, the total levels of histone acetylation are likely to change as the organism ages. Consistent with this observation, the levels of the histone deacetylase HDAC-1 decrease upon serial passaging of primary human fibroblasts [34]. Importantly, treatment of primary human fibroblasts with the HDAC inhibitors Trichostatin A (TSA) or sodium butyrate induce a senescence-like state, suggesting that modulation of histone acetylation through class I and II HDACs is an essential step in the establishment of senescence [35]. Whether this effect results from global changes in histone acetylation or from the transcriptional activation of discrete loci remains unknown. Seemingly contradictory, the recent observation that HDAC1 overexpression in melanoma cells is sufficient to induce an irreversible senescence program demonstrates that the function of class I HDACs in senescence is likely to be more complex than initially anticipated [36]. The precise contribution of HDAC1 and other class I and II HDACs to the establishment and maintenance of the senescent state and organismal aging will undoubtedly become more clear in the near future as genome wide approaches, including promoter location analysis, allow the delineation of HDACs targets upon pro-senescence signals.

### The complex case of sirtuins

The members of the evolutionary conserved Sirtuin family of proteins are nicotinamide adenine dinucleotide (NAD)-dependent proteins with histone deacetylase activity, whose founding member is the yeast Sir2 protein. In yeast, worms and flies, extra copies of sirtuins extend replicative lifespan, and the deacetylase enzymatic activity of sirtuins is required for this function [37-40]. Additionally, Resveratrol, a potent inducer of sirtuins enymatic activity, extends life span in these species, in a sirtuin-dependent manner [41]. The correlation between sirtuins and aging in metazoans has led to an interest in the mammalian homologs of Sir2, the SIRT proteins. The mammalian SIRT family consists of seven members, SIRT1-7. SIRT1, however, is reported to be the most similar to yeast Sir2. The contribution of SirT1 to senescence and aging in mammals is complex, and may involve non-chromatin substrates. For instance, SIRT1 was shown to deacetylate p53 *in vivo*, leading to p53 inactivation [42-44]. In addition, loss-of-function experiments in mouse fibroblast demonstrated that SIRT1 is required for replicative senescence resulting from chronic genotoxic stress [42]. In these experiments, SIRT1-/- mouse embryonic fibroblasts (MEFs) fail to upregulate p19^ARF^ upon serial passaging in normal culture conditions, correlating with their inability to undergo replicative senescence. Whether this effect of SIRT1 on p19^ARF^ regulation involves chromatin regulation or involves the deacetylation of a non-histone protein is unknown. Recently, a direct correlation between SIRT1 regulation of chromatin structure and aging has been uncovered in mouse embryonic stem cells [45]. Using genome-wide location analysis, it was demonstrated that, upon genotoxic stress, SIRT1 is delocalized from repeat sequences and numerous gene promoters to bind to sites of DNA damage, in a process consistent with the afore-mentioned "RCM" hypothesis (for redistribution of chromatin modifiers). Such relocalization may affect the aging process in a dual manner: First, it may suppress genomic instability by contributing to DNA repair upon chronic genotoxic stress that occurs in aging organisms. Second, the loss of SIRT1-driven transcriptional repression at its natural targets correlates with the deregulation of gene expression consistent with what is observed in aging tissues [45]. These observations strongly suggest that forced expression of SIRT1 may represent a strategy to alter aging-related chromatin changes in mammals.

Another mammalian sirtuin family member, the SIRT6 protein, has been involved in aging-related chromatin changes in mice. SIRT6 deficient mice display a premature aging-phenotype and die rapidly after birth, displaying an acute multi-organ degenerative syndrome [46]. While SIRT6 has been shown to prevent telomere dysfunction in human cells by deacetylating H3K9 at telomeric loci (see below), this function is not conserved in mouse cells and therefore cannot account for the aging-phenotype in SIRT6-/- mice [47]. A recent report shed light on a potential molecular mechanism for SIRT6-mediated prevention of aging in mice [48]: SIRT6 was shown to be tethered by NF-κB target genes and to deacetylate H3K9 on these promoters, thus attenuating this signaling pathway. Since NF-κB has been implicated in the induction of a senescence specific program and genetic alteration of NF-κB signaling rescues the aging phenotype elicited by SIRT6 deficiency [48,49], it is tempting to conclude that SIRT6 prevents aging at least partly through the modulation of the chromatin structure and transcription program driven by this specific signaling pathway. Finally, reinforcing the connection between DNA damage and cellular senescence, SIRT6 was recently shown to deacetylate H3K9 at sites of double strand breaks (DSB), and to be required for the mobilization of DNA-PK at these sites and the subsequent resolution of DSBs [50].

### Senescence Associated Heterochromatic Foci and the induction of senescence

As presented above, senescence, along with stem cell depletion, is one cellular manifestation of organismal aging. A direct involvement of chromatin modifications in the establishment of the senescent phenotype was recently revealed in that senescent cells display specialized and discrete subnuclear structures called S enescence A ssociated H eterochromatic F oci (SAHF) [51]. SAHF were first described in human primary fibroblasts driven to senescence via serial passaging or oncogenic stress [52]. Importantly, these heterochromatic foci are not present in quiescent cells. SAHF are easily stained in senescent human cells upon incubation with 4'-6-Diamidino-2-phenylindole staining (DAPI), resulting in the visualization of bright positive nuclear foci. These foci are resistant to digestion by nucleases, consistent with the dense heterochromatic nature of the corresponding genomic loci. Indeed, SAHF contain chromatin marks that are reminiscent of those found in constitutive heterochromatin, including hypoacetylated histones, methylation of lysine 9 of histone H3, and Heterochromatin Protein 1 (HP1) [52]. However, SAHF also contain specific marks, absent from constitutive heterochromatin such as enrichment of macroH2A and HMGA proteins and depletion of linker histone H1 [53-55]. Consistent with a function in the permanent cell cycle exit upon induction of senescence, SAHF embed genomic loci encoding pro-proliferative proteins into heterochromatin structures, thus preventing their accessibility by the transcription machinery [52]. In addition, it appears that each chromosome condenses into one single SAHF, where the loci to be repressed are found in the interior or immediate periphery of the corresponding focus [55]. The molecular events leading to the formation of SAHF are still under investigation, but several proteins have been demonstrated to contribute to the formation of SAHF: They include the histone chaperones HIRA and Asf1, HP1g, HMGAproteins, and the H3K9me3 methyl-transferases Suv39h1/h2 [54,56,57]. Impor-tantly, genetic disruption of Suv39h1 in mouse lymphocytes promotes tumorigenesis by preventing oncogene-induced senescence upon activated Ras overexpression [56] This important result suggests that the formation of SAHF is essential for senescence to occur *in vivo*. However, it is important to note that primary fibroblasts inactivated for Suv39h1 and h2 remain susceptible to replicative senescence [52,58]. This discrepancy suggests that either the formation of SAHF upon replicative or oncogene signal utilize different pathways, or that the enzymatic machinery required for SAHF formation differs in lymphocytes and in fibroblasts. More experiments are needed to precisely define the contribution of SAHF to the senescent phenotypes. In addition, many questions regarding the generation of these specialized loci remain, including the identity of the complex required for the deacetylation of the loci embedded in SAHF, as well as the molecular basis for the targeting and coordination of the different chromatin modifying activities affecting SAHF formation. Finally, since all chromatin modifications identified in SAHF have now been shown to be reversible, the irreversible nature of senescence is likely to rely on additional molecular mechanisms yet to be identified.

### Lamins, progeria and the aging phenotype

Lamins belong to a family of intermediate filaments, which are the main structural components of the nuclear lamina. They have been shown to play a role in chromosome organization and to interact with chromatin and DNA through lamin binding proteins [59]. Importantly, lamins are mutated in multiple human diseases, known as laminopathies. The connection between lamins and aging was first suggested through the genetic analysis of a progeria syndrome: Hutchinson-Gilford progeria syndrome (HGPS) is a rare genetic disorder characterized by premature aging. Patients are born normal, but develop age-associated disorders within a year after birth. Characteristic symptoms include hair loss, artheriosclerosis, loss of subcutaneous fat, growth retardation, osteoperosis, and aged skin [60]. HGPS is caused by a single nucleotide substitution in the Lamin A gene resulting in a cryptic splice site in exon 11 causing a 150 nucleotide-deletion from the 3' end of the lamin A gene, leading to the synthesis of an abnormal protein, LA∆50 [61,62]. Fibroblasts from young HGPS patients have relatively normal nuclei and nuclear lamina. However, as these cells are passaged in culture, several abnormalities become detectable in the nucleus. These include lobulation of the nuclear envelope, thickening of the nuclear lamina, clustering of nuclear pores, and loss of peripheral heterochromatin [59]. The severity of these nuclear abnormalities is highly correlated with an overall accumulation of the LA∆50 protein upon passage in culture [63]. Importantly, ectopic expression of LAΔ50 protein in normal cells is sufficient to recapitulate the nuclear defects observed in cells derived from HGPS patients [63]. Further strengthening the connection with cellular aging is the recent observation that similar mechanisms and defects occur in healthy individuals. Indeed, fibroblasts from older healthy individuals (81 to 96 years) express the LA∆50 transcript at low levels and display nuclear defects similar to those from HGPS patients [64].

At the chromatin level, HGPS fibroblasts exhibit a loss of nuclear peripheral heterochromatin, and, in cells derived from older HGPS patients, several heterochromatin marks, including mono-methylated H3K9 and tri-methylated H3K9, are globally almost undetectable. As female HGPS fibroblasts are passaged in culture, a reduction of H3K27 trimethylation is observed on the inactive X chromosome. In fact, loss of this mark was detected in early passage cultures before any nuclear defects were observed suggesting heterochromatin defects precede nuclear defects [27]. The loss of the H3K27 tri-methyl mark was also correlated with a 9-10 fold decrease in EZH2, the enzyme responsible for maintenance of this mark [27]. Furthermore, late-passage HGPS cells show an upregulation of H4K20 tri-methyl [27], which, as presented above, is also observed in livers of older rats [26]. Importantly, similar defects in heterochromatinization accumulate, though to a lesser extent, in aging wild-type cells. Specifically, skin fibroblasts from healthy old individuals (81-96 years) show decreased staining for HP1 and H3K9me3 compared to the same cells from young individuals (3-11 years) [64].

The molecular mechanisms linking lamin A to heterochromatin remains unclear, but two hypothesis are currently being explored. First, the function of specific transcription factors could be affected by mutations in Lamin A. One candidate is the retinoblastoma protein, Rb, which binds directly to type-A lamins and whose function is regulated by this association [65,66]. Given the role of Rb in the regulation of histone methylation [67], including H3K27, H3K9 and H4K20, it is conceivable that alteration of Lamin A affects histone methylation through impairment of the Rb pathway. A second, non-exclusive, hypothesis is that defects in Lamin A protein induce changes in nuclear localization of specific loci. For example, chromosome territories can be displaced from the nuclear periphery to the nuclear interior upon expression of mutant Lamin A. This would be consistent with the recent demonstration that Lamins associated microenvironments are organized into transcriptionally defined domains [68]. Given the connection between nuclear tethering and transcription/chromatin modifications [69], alterations in nuclear envelope structure resulting from Lamin A mutations are likely to affect chromatin globally. Although still correlative, these studies strongly suggest that loss of heterochromatin in the HGPS model could potentially drive the premature aging process and may contribute to the normal aging process.

### Chromatin structure of telomeres and the aging phenotype

Telomeres are repetitive DNA elements at the tips of chromosomes that protect DNA ends from recom-bination and degradation [70]. As mammals age, their telomeres become shorter during each round DNA replication due to the inability of the transcription machinery to completely replicate the ends. Eventually, human telomeres reach a critically short length that activates the DNA damage pathway leading to replicative senescence. Telomere attrition is considered to be a mechanism for organismal aging in which shortened telomeres lead to stem cells depletion and eventual loss of tissue regeneration [71].

Telomeres from normal mouse lab strains are much longer than those from humans and therefore, do not erode enough to induce senescence. However, studies in mice have allowed elucidation of the mechanisms behind epigenetic regulation of telomeres, which are likely to be relevant in other mammalian species. These studies demonstrate that telomeres are heterochromatic in nature, and that loss of heterochromatic marks may regulate telomere length, thus potentially affecting cellular aging. Specifically, subtelomeric regions comprise methylated DNA, as well as H3K9 and H3K20 methylated nucleosomes [72,73]. Loss of DNA methyltransferase activity in mouse embryonic stem cells results in decreased methylation at subtelomeric regions hence increased telomere recombination [74]. Moreover, MEFs deficent in the Suv39h1 and Suv39h2, H3K9 histone methyltransferases, have abnormally long telomeres, in agreement with the hypothesis that chromatin structure contributes to the regulation of telomere length [72]. In the telomerase deficient mouse, critically short telomeres are characterized by the loss of heterochromatin marks at subtelomeric regions including DNA methylation, H3K9 and H4K20 trimethylation and histone hypoacetylation [75]. Therefore, the interplay between chromatin modifications and telomere length appears to be complex and remains to be fully investigated.

 Recent studies in human cells have directly linked chromatin modifications to cellular senescence. Human primary fibroblasts, in which levels of the H3K9 deacetylase SIRT6 are experimentally decreased, undergo premature cellular senescence [47]. These cells exhibit telomere dysfunction and chromosomal end-to-end to fusions, specifically linked to alteration of SIRT6 enzymatic activity. SIRT6 knockdown human fibroblasts and SIRT6 deficient mouse cells show H3K9 hyperacetylation at telomeric loci, thereby, demonstrating SIRT6 H3K9 deacetylase targets telomeres [47]. This study demonstrates that telomere length and function require epigenetic modifications and alterations to these regulatory mechanisms lead to telomeric dysfunction and a subsequent senescent phenotype

### Concluding remarks

As organisms age, changes occur at many levels, including transcriptional regulation and nuclear architecture. While it becomes clear that modulation of the chromatin structure influences molecular events at the nexus of cellular aging, several questions remain: First, are specific chromatin modifications a cause or a consequence of cellular senescence? As presented above, loss-of-function experiments have begun to shed light on the direct contribution of specific modifiers to cellular aging. Second, if chromatin modifiers can directly contribute to the aging phenotype, what is the molecular circuitry leading to the modulation of their activities during the aging process, and may it be altered as a therapeutic means? Finally, one of the biggest challenges in the understanding of the aging process is to decipher the connections between the seemingly independent molecular events that have been reported in different settings of senescence. The recent development of small molecules that interfere with specific histone modifiers and their use in clinical trials, should provide new opportunities for the therapeutic modulation of the aging phenotypes in the future.
